# Clinical Reasoning Needs to Be Explicitly Addressed in Health Professions Curricula: Recommendations from a European Consortium

**DOI:** 10.3390/ijerph182111202

**Published:** 2021-10-25

**Authors:** Ioannis Parodis, Lina Andersson, Steven J. Durning, Inga Hege, Jure Knez, Andrzej A. Kononowicz, Marie Lidskog, Tadej Petreski, Magdalena Szopa, Samuel Edelbring

**Affiliations:** 1 Division of Rheumatology, Department of Medicine Solna, Karolinska Institutet and Karolinska University Hospital, 171 76 Stockholm, Sweden; 2 Department of Rheumatology, Faculty of Medicine and Health, Örebro University, 702 81 Örebro, Sweden; 3 School of Health Sciences, Örebro University, 702 81 Örebro, Sweden; lina_andersson@ymail.com (L.A.); samuel.edelbring@oru.se (S.E.); 4 Department of Medicine, Uniformed Services University of the Health Sciences, Bethesda, MD 20814, USA; steven.durning@usuhs.edu; 5 Medical Education Sciences, Medical School, University of Augsburg, 86159 Augsburg, Germany; inga.hege@med.uni-augsburg.de; 6 Division for Gynaecology and Perinatology, University Medical Centre Maribor and Faculty of Medicine, University of Maribor, 2000 Maribor, Slovenia; jure.knez1@um.si; 7 Department of Bioinformatics and Telemedicine, Jagiellonian University Medical College, 30 688 Krakow, Poland; andrzej.kononowicz@uj.edu.pl; 8 School of Medical Sciences, Örebro University, 702 81 Örebro, Sweden; marie.lidskog@oru.se; 9 Department of Nephrology, Clinic for Internal Medicine, University Medical Centre Maribor and Faculty of Medicine, University of Maribor, 2000 Maribor, Slovenia; tadej.petreski1@um.si; 10 Department of Medical Education, Jagiellonian University Medical College, 30 688 Krakow, Poland; magdalena.szopa@uj.edu.pl; 11 Department of Metabolic Diseases, Jagiellonian University Medical College, 30 688 Krakow, Poland

**Keywords:** clinical reasoning, curriculum development, curriculum mapping, health professions education, medical education

## Abstract

Clinical reasoning entails the application of knowledge and skills to collect and integrate information, typically with the goal of arriving at a diagnosis and management plan based on the patient’s unique circumstances and preferences. Evidence-informed, structured, and explicit teaching and assessment of clinical reasoning in educational programs of medical and other health professions remain unmet needs. We herein summarize recommendations for clinical reasoning learning objectives (LOs), as derived from a consensus approach among European and US researchers and health professions educators. A four-step consensus approach was followed: (1) identification of a convenience sample of the most relevant and applied national LO catalogues for health professions educational programs (N = 9) from European and US countries, (2) extraction of LOs related to clinical reasoning and translation into English, (3) mapping of LOs into predefined categories developed within the Erasmus+ Developing, implementing, and disseminating an adaptive clinical reasoning curriculum for healthcare students and educators (DID-ACT) consortium, and (4) synthesis of analysis findings into recommendations for how LOs related to clinical reasoning could be presented and incorporated in LO catalogues, upon consensus. Three distinct recommendations were formulated: (1) make clinical reasoning explicit, (2) emphasize interprofessional and collaboration aspects of clinical reasoning, and (3) include aspects of teaching and assessment of clinical reasoning. In addition, the consortium understood that implementation of bilingual catalogues with English as a common language might contribute to lower heterogeneity regarding amount, structure, and level of granularity of clinical reasoning LOs across countries. These recommendations will hopefully motivate and guide initiatives towards the implementation of LOs related to clinical reasoning in existing and future LO catalogues.

## 1. Introduction

Curriculum development for health professions education (HPE) is commonly guided by overarching collections of learning objectives (LOs). However, these are often disconnected from explicit objectives for the core ability of clinical reasoning [1,2]. While varying definitions exist [3], clinical reasoning has been defined as the cognitive and non-cognitive process by which a health professional consciously and unconsciously interacts with the patient and the environment to collect and interpret data, weigh the benefits and risks of actions, and understand the patient preferences to determine a diagnostic and therapeutic management plan with the purpose to improve the patient’s well-being [4]. Clinical reasoning towards decision making may occur at micro (e.g., tissue, cellular, and molecular level), macro (e.g., symptoms and signs), and meta (synthesis) levels, and it may be individually or collaboratively conducted [5]. Due to its multifactorial nature and unconscious components, clinical reasoning is not only difficult to learn, but also difficult to teach in a systematic manner. Overlooking clinical reasoning in formal education necessitates that training of this ability occurs in the form of informal activities in clinical practice. Although clinical reasoning ability develops to varying extents within clinical practice, this cannot assure that all aspects of this compound ability have been accounted for. Failure to achieve an adequate level of clinical reasoning ability constitutes a major threat to patient safety, potentially leading to negative consequences for the patients and society, including inappropriate treatment and medical procedures, and suboptimal use of societal resources. Hence, structured and explicit evidence-informed teaching and assessment of clinical reasoning in educational programs of health professions constitute important needs [2,6].

In many countries, LO collections have been developed for the educational programs of medical and other health professions, with the purpose to serve as a blueprint throughout the study programs. Such often termed LO “catalogues” interpret and detail the requirements for HPE posed by national and local legislation and provide guidance for curriculum developers when reforming a curriculum or developing a new study program. Additionally, LO catalogues can be used for curriculum mapping purposes [7] and to facilitate communication about and comparison of curricula within and across different health professions educational programs.

Towards the goal of conceptualizing, developing, evaluating, and disseminating a clinical reasoning curriculum for students and educators, we herein summarize recommendations for LOs to strengthen explicit support for teaching and assessment of clinical reasoning, as derived from a consensus approach among health professionals, researchers, and educators from Europe and the US within the Erasmus+ Developing, implementing, and disseminating an adaptive clinical reasoning curriculum for healthcare students and educators (DID-ACT) consortium.

## 2. Materials and Methods

For the development of the recommendations, DID-ACT partner universities and stakeholders from seven countries (Germany, Malta, Poland, Slovenia, the USA, Sweden, Switzerland) analyzed LO catalogues developed to guide learning within different health professions [8]. The data were collected and analyzed during workshops involving multiple professions, competencies, and perspectives.

The consensus process followed a four-step approach. Firstly, we determined a convenience sample of the most relevant and most applied national LO catalogues for health professions educational programs from the DID-ACT partner countries and contexts. This selection was based on partners’ experience in clinical reasoning research and teaching, review of the literature, and internet search. Each partner provided information about the characteristics and content of their national catalogues.

In separate analyses of each one of these catalogues, we identified, extracted, and collected LOs relating to clinical reasoning; those LOs were subsequently translated into English. In a third step, we mapped the LOs and stratified them into predefined categories. These categories had previously been developed by the consortium with the purpose of identifying core areas and potential gaps across LO catalogues [9], and comprised (1) theories of clinical reasoning, (2) gathering, interpreting, and synthesising patient information, (3) generating differential diagnoses including defining and discriminating features, (4) developing a treatment/management plan, (5) aspects of patient participation in clinical reasoning, (6) collaborative aspects of clinical reasoning, (7) interprofessional aspects of clinical reasoning, (8) ethical aspects, (9) self-reflection on clinical reasoning performance and strategies for future improvement, (10) errors in the clinical reasoning process and strategies to avoid them, (11) attitudes towards clinical reasoning teaching, (12) teaching, assessing, and evaluating clinical reasoning, and (13) decision making. We also conducted a comparison across catalogues with respect to their structure and identified the parts/chapters of the catalogues where LOs related to clinical reasoning were represented. Finally, we summarized the findings from the analyses during consensus meetings and drafted recommendations for how LOs related to clinical reasoning could be effectively presented and incorporated in LO catalogues. The final recommendations were discussed and agreed upon by all DID-ACT partners. During a parallel work within the DID-ACT consortium, a set of learning goals and objectives specifically addressing clinical reasoning was synthetized and consensually agreed upon to serve as a guide for future adoption into curricula.

## 3. Results

### 3.1. Overview of National LO Catalogues

We identified and included eight LO catalogues from three professions (medicine, nursing, physiotherapy), applied within HPE in Sweden, Poland, Germany, Switzerland, and the USA as of 31 December 2020. Additionally, we included results from the EU-funded project “TUNING Educational Structures in Europe” [10]. An overview of these LO catalogues is presented in Table 1.

We could not identify any national LO catalogues for Slovenia or Malta. In these countries, each school defines the LOs within the respective study program. The physiotherapy education in Germany is currently based on a national training and examination framework [11], which does not provide LOs but a list of topics that have to be accounted for in curricula across programs.

The catalogues identified for analysis did not include LOs for the purpose of training of health professionals in their role as teachers (train-the-trainer LOs). In many European countries, a certain didactical qualification of health professionals prior to teaching is mandatory [12], but the frameworks that are available for this kind of faculty development within health professions are characterized by a high level of abstraction. In Table 2, we present exemplary programs and concepts for faculty development at a national and international level.

Notably, none of the programs presented in Table 2 explicitly addressed clinical reasoning. In addition, in the interviews that we conducted as a part of our preceding analysis of needs, all respondents indicated that there was no faculty development program or course at their institution that was specifically dedicated to teaching of clinical reasoning [1]. For this reason, no catalogues addressing faculty development were included in the analysis.

### 3.2. Analysis of LOs in Catalogues

We analyzed how the different LOs in the catalogues related to the LO categories developed by the consortium [9]. We found that some categories were represented in nearly all catalogues; those included “Gathering, interpreting, and synthesising patient information” and “Developing a treatment/management plan”. Other categories were not covered at all or were included in only few catalogues; those included “Theories of clinical reasoning” and “Attitudes towards clinical reasoning”. Additionally, interprofessional aspects were mostly covered in nursing catalogues, with little coverage in catalogues within medicine, with the exception of Principal Relevant Objectives and Framework for Integrative Learning and Education in Switzerland (PROFILES) [13]. Table 3 outlines the number of LO catalogues and LOs, mapped into facets of clinical reasoning, according to the aforementioned categories that were developed within the DID-ACT project.

The category “Gathering, interpreting, and synthesising patient information” was the one covered most extensively. Examples included (1) “synthesise essential data from previous records, integrate the information derived from history, meaningful physical and mental symptoms and physical examination; provide initial diagnostic evaluations; take into account the age, gender and psychosocial context of the patient, as well as social determinants of health” (PROFILES); (2) “graduates in medicine will have the ability to order appropriate investigations and interpret the results” (TUNING); and (3) “the trainees collect care-related data from people of all ages with health problems and related resources and resistance factors” (Framework for theoretical and practical teaching in nursing).

Examples from the category “Developing a treatment/management plan” included the following LOs: (1) “the student is able to demonstrate knowledge of the planning, management and coordination of healthcare measures” (The Higher Education Ordinance); (2) “in a patient, identify conditions that require urgent treatment and establish and initiate an initial treatment plan in consultation with the patient and/or relatives” (Entrustable Professional Activities, EPA; Sweden); and (2) “choose a treatment that minimises the social consequences for the patient” (Polish Ministry of Science and Higher Education Educational Outcomes for Health Professions Catalogue).

The topics that were most prominent in the LO catalogues, i.e., “gathering, interpreting, and synthesising patient information”, “developing a treatment/management plan”, and “generating differential diagnoses”, were also the most prominent aspects emerging from our analysis of needs [1]. Similarly, topics that were less prominent in the LO catalogues, e.g., “theories of clinical reasoning”, were also less solicited for in the DID-ACT analysis of needs, as described in earlier research and project reports [1,2].

### 3.3. Coverage of LOs Related to Clinical Reasoning in LO Catalogues

Detailed results from the mapping of LOs related to clinical reasoning in the different chapters of LO catalogues are provided in the accompanying Supplementary Table S1. It is worth noting that the heterogeneous structure across catalogues made the comparison across catalogues burdensome.

### 3.4. Recommendations

We developed three recommendations that specifically address how clinical reasoning could be embodied in LO catalogues and at a curricular level. We also identified assets addressing the need to circumvent communication barriers across universities, cultures, and contexts. Upon analysis of the LO catalogues, as described above, we discussed and agreed upon the recommendations within the consortium. We also highlighted how these recommendations are related to barriers for introducing a clinical reasoning curriculum that was previously identified by the consortium [1], and developed a set of overarching principles and exemplary goals and objectives to facilitate future adaptation in curricula. The recommendations are summarized in Box 1 and are described in detail in the following subchapters. An initial project report was made available online by the DID-ACT consortium [14].

Box 1 **Recommendations for learning objectives related to clinical reasoning.**

Make clinical reasoning explicit

- Subsume learning objectives related to clinical reasoning.
- Flag or tag learning objectives that are related to clinical reasoning in curriculum mapping processes.
- Include learning objectives that are related to key theories of clinical reasoning.

Emphasise interprofessional and collaboration aspects of clinical reasoning

- Include communication, collaboration, and interprofessional aspects of clinical reasoning in health professions curricula.
- Make explicit communication, collaboration, and interprofessional aspects of clinical reasoning in learning objective catalogues.

Include aspects of teaching and assessment of clinical reasoning

- Emphasise the role of educators in the acquisition of clinical reasoning ability.
- Include and make explicit learning objectives for educators in learning objective catalogues.

Generic assets to alleviate barriers

- Implementation of bilingual curricula with English as a common language is advisable in order to facilitate comparisons and alleviate the heterogeneity in the amount, structure, and level of granularity of clinical reasoning learning objectives across educational programmes and countries.

#### 3.4.1. Make Clinical Reasoning Explicit

Based on experience and research, we acknowledge that clinical reasoning is a complex ability, which is challenging to conceptualize as a precise learning outcome for students. However, this should not hinder efforts to aim for explicit teaching of clinical reasoning. Thus, our first recommendation is that clinical reasoning should be made explicit in LO catalogues. Examples of how this recommendation could be implemented in LO catalogues include (1) subsuming LOs related to clinical reasoning, which could be presented in a chapter explicitly dedicated to clinical reasoning, (2) flagging or tagging LOs that are related to clinical reasoning in curriculum mapping processes, and (3) incorporating LOs that are related to key theories of clinical reasoning. The last could also make teaching of clinical reasoning more explicit.

#### 3.4.2. Emphasize Interprofessional and Collaboration Aspects of Clinical Reasoning

Our analysis showed that training in collaboration and interprofessional aspects of clinical reasoning was only included in LO catalogues for nursing education. An increasingly high degree of specialisation in healthcare and the increasing multi-morbidity due to the demographic development towards elderly populations require communication and collaboration across health professions in a person-centered approach. Therefore, collaboration and interprofessional aspects of clinical reasoning should be included in all health professions curricula, and we recommend that this is made explicit in LO catalogues. Examples of such LOs could include that the student (1) will be able to make use of team competencies regarding patient information and diagnostic and therapeutic management, (2) will be able to collaborate and communicate across professions in the clinical reasoning process to meet patient needs, (3) will understand how personal, professional, and interprofessional values affect interprofessional care, and (4) will understand similarities and differences in clinical reasoning across health professions. Additionally, our analysis highlighted the importance of patient involvement in the clinical reasoning and decision-making processes [9].

#### 3.4.3. Include Aspects of Teaching and Assessment of Clinical Reasoning

Teaching aspects of clinical reasoning are neither explicitly included in the faculty development frameworks (Table 2) nor in the LO catalogues (Table 3), despite the agreed upon importance and relevance of this aspect. Some of the LO catalogues, such as the National Competency-based Catalogue in Medicine (NKLM) and PROFILES, are based on the CanMEDS framework, including LOs for the role as a “scholar”. To also facilitate teaching of clinical reasoning and how the intended learning outcomes can be achieved and assessed, we recommend that LOs specifically addressing aspects of teaching and assessment of clinical reasoning are added to these catalogues. For example, an important LO for train-the-trainer courses could read “the learner will be able to choose appropriate teaching, assessment, and evaluation methods for clinical reasoning and adapt those to the cultural context”.

This recommendation also links back to our first recommendation of making clinical reasoning explicit, putting additional emphasis on the teacher and assessor level. Importantly, this also addresses the lack of awareness of the fact that clinical reasoning can be taught, a barrier that we identified in the preceding analysis of needs within the DID-ACT consortium [1].

#### 3.4.4. General Assets to Alleviate Barriers

We discovered that talking about curricula and exchanging experiences across partners and contexts was challenging for multiple reasons. Firstly, the different languages of the catalogues since LO catalogues are mainly available only in the respective national language. Secondly, the structure and level of granularity of the LO catalogues were highly heterogeneous. The number of LOs that were related with clinical reasoning in the different catalogues ranged from 4 to 22, and those displayed different levels of granularity, making comparisons and in-depth analyses challenging. Implementation of bilingual catalogue versions, e.g., with English as a common language, was identified as an action with potentiality to facilitate increased communication across policy makers within health professions schools, both with regard to goals about clinical reasoning and how those are formulated in local contexts. Subsequently, increased communication across educators could result in gradually lower heterogeneity regarding amount, structure, and level of granularity of LOs related with clinical reasoning.

### 3.5. Developing a Set of Clinical Reasoning Goals and Objectives

In a parallel work, the consortium agreed upon overarching principles and a set of learning goals and objectives that constitute examples of LOs related to clinical reasoning that could be incorporated in educational programs, and categorized them into LOs for student curricula and LOs for educators, e.g., within train-the-trainer courses. Those examples were further subcategorized into the aforementioned DID-ACT categories, and are presented in Box 2; the initial version of this work was made accessible online by the consortium [9].

Box 2 **Learning goals and objectives of clinical reasoning.**

**Learning objectives for clinical reasoning student curricula**

*Overarching goal: to increase health professions students’ awareness of and skills in clinical reasoning.*

Gathering, interpreting, and synthesising patient information

- The student will be able to accurately and efficiently collect key clinical findings needed for analysis of the patient’s problem.
- The student will be able to accurately and efficiently analyse and interpret the key clinical findings to plan patient treatment and care.

Reasoning towards the development of a management plan

- The student will be able to apply treatment, therapeutic and prophylactic procedures based on a holistic assessment of the patient, the diagnosis, the healthcare context, alongside with current scientific evidence.
- The student will know how to set treatment goals for the patient based on evidence, healthcare context, and patient needs and preferences.

Aspects of patient participation in clinical reasoning

- The student will be able to engage and collaborate with patients and families in the diagnostic process and analysis of the patient’s problem, in accordance with their values and preferences.
- The student will be able to involve and support the patient in a shared decision-making process about the management plan.

Collaborative aspects of clinical reasoning

- The student will be able to make use of team competencies regarding patient information, and diagnostic and therapeutic management.

Self-reflection on clinical reasoning performance and strategies for future improvement

- The student will know how to use self-reflection and critical thinking to improve diagnostic, therapeutic, and disease management performance.
- The student will be able to evaluate the outcomes of the clinical reasoning and plan for appropriate improvements together with patients and colleagues.

Generating differential diagnoses including defining and discriminating features

- The student will be able to generate differential diagnoses, including defining and discriminating features.
- The student will demonstrate an understanding of the benefits and risks of using clinical decision support systems, including artificial intelligence in clinical reasoning.

Cognitive errors and bias in the clinical reasoning process and strategies to avoid them

- The student will develop an understanding of the benefits of open climate that allows sharing of reasoning errors, thus promoting continuous learning and patient safety.
- The student will be able to explain the occurrence of uncertainty in the clinical reasoning process under different circumstances and how to deal with it in a safe manner.
- The student will be able to demonstrate an understanding of how emotions can influence clinical reasoning.
- The student will be able to overcome common challenges and errors during the clinical reasoning process.

Ethical aspects

- The student will be able to take legal, moral, diversity, gender-related, and ethical aspects into account in the clinical reasoning process.

Interprofessional aspects of clinical reasoning

- The student will be able to collaborate and communicate across professions in the clinical reasoning process to meet patient needs.
- The student will be able to demonstrate an understanding of how personal, professional, and interprofessional values affect interprofessional care.
- The student will be able to demonstrate an understanding of similarities and differences in clinical reasoning across health professions.

Understanding of key theoretical models related to clinical reasoning

- The student will be able to relate key theories and models of clinical reasoning, e.g., illness scripts, pattern recognition, and dual theory, to clinical practice.

**Learning objectives of clinical reasoning for educators**

*Overarching goal: to develop skills in teaching and assessing clinical reasoning in health professions educational programmes.*

(Interprofessional) collaboration and exchange

- The learner will be able to successfully teach about similarities, differences, and the most common sources of errors in the clinical reasoning process across health professions.

Awareness of and critical reflection on the importance of learning and teaching clinical reasoning

- The learner will be motivated and inspired to teach and assess clinical reasoning.
- The learner will develop an awareness of and openness to share errors in clinical reasoning teaching.
- The learner will develop an awareness of why and how reasoning errors can be used in clinical reasoning teaching.

Teaching, assessing, and evaluating clinical reasoning

- The learner will be able to implement aspects of the clinical reasoning student curriculum in their teaching.
- The learner will be able to use various methods of assessing clinical reasoning in relation to specific needs.

Patient-related aspects

- The learner will be able to teach and assess patient involvement in clinical reasoning.

The clinical reasoning LOs for adaptation in student curricula are guided by the overarching goal of increasing health professions students’ awareness of and ability in clinical reasoning and cover objectives of competence in gathering, interpreting, and synthesising information, skill to reason towards a management plan, patient involvement, collaborative and ethical aspects, and awareness of theoretical models, as well as self-reflection on performance and strategies for future improvement. With the overarching goal of developing skills for teaching and assessing clinical reasoning in the education of health professionals, the second part of this set of LO examples is directed to educators. This covers objectives of collaboration and exchange, awareness of and critical reflection on the importance of learning and teaching clinical reasoning, and ability to choose appropriate teaching, assessment, and evaluation methods, as well as adapting those into the respective context. Last but not least, the importance of patient involvement is emphasized [9].

## 4. Discussion

Several articles and recommendations for LO development and curriculum mapping [7,15,16,17] have been published. Taxonomies, e.g., Bloom’s Taxonomy [18], the constructive alignment theory [19], and guidelines on how to draft LOs, e.g., the SMART approach [20], support educators in formulating LOs and aligning them with the curriculum. In the present work, we analyzed eight LO catalogues from three health professions applied within different educational programs in Sweden, Poland, Germany, Switzerland, and the USA. Notably, the core ability of clinical reasoning was not explicitly addressed, nor was its teaching and assessment in an overview of faculty development programs and frameworks. With the purpose of contributing to conceptualization, development, evaluation, and dissemination of a clinical reasoning curriculum for students and educators, we developed three additional distinct recommendations, i.e., (1) to make clinical reasoning explicit in national LO catalogues, (2) to emphasize interprofessional and collaboration aspects of clinical reasoning, and (3) to include aspects of teaching and assessment of clinical reasoning. Additionally, the consortium reckoned that implementation of bilingual curricula with English as a common language is advisable. Along with these recommendations, we herein also provided a set of overarching principles and learning goals and objectives for clinical reasoning curricula for both students and educators, as agreed upon within a consortium of researchers and health professions educators across six European countries and the US.

The recommendation of making clinical reasoning explicit acknowledges that clinical reasoning is a complex ability to acquire and, therefore, hard to conceptualize in LOs. We provided examples of how clinical reasoning can be made explicit in LO catalogues, i.e., by subsuming and flagging relevant LOs in catalogues or including LOs related to selected basic theories of clinical reasoning. Making clinical reasoning explicit in LO catalogues can support exchange of knowledge and communication of expertise with regard to this core ability among educators and implicitly addresses specific barriers that were previously identified by the consortium [1], i.e., (1) the lack of awareness of clinical reasoning, (2) the lack of awareness that clinical reasoning can be taught, and (3) the lack of standards for the teaching of clinical reasoning. In the long term, making clinical reasoning more explicit in LO catalogues can be a first step towards providing longer and dedicated curricular time for teaching clinical reasoning, as recently attempted with the creation of a longitudinal clinical reasoning curriculum within a medical school program in the United Kingdom [21].

With regard to the second recommendation, we foresee that making collaboration and interprofessional aspects of clinical reasoning explicit would help address cultural barriers in clinical reasoning curricula [1], especially communication issues across health professions and profession-specific perspectives on clinical reasoning. This is a well-known barrier, identified in research within our consortium [22] as well as by others in previous research [6,15].

Our third recommendation addresses that teaching of clinical reasoning is not made explicit in current faculty development frameworks or LO catalogues, despite its importance and relevance. It further acknowledges that the addition of LOs addressing clinical reasoning in LO catalogues may not be sufficient unless such additions are complemented by LOs addressing how achievement and assessment of the intended learning outcomes can be facilitated, e.g., in train-the-trainer courses. Thus, LOs that explicitly address teaching and assessment of clinical reasoning would also indirectly support the first and most important recommendation presented in the current work, i.e., making clinical reasoning explicit, putting emphasis on the teacher perspective. Furthermore, we foresee that implementation of this recommendation would result in increased awareness among students and teachers of the fact that clinical reasoning can be taught, which we identified as a currently unmet need.

Along with the aforementioned recommendations that are expected to specifically foster contemplation of clinical reasoning in LO collections, some general points were also derived from the analysis, in particular the need of addressing hurdles posed in international communication and exchange of experiences across universities around the globe. The heterogeneity in the structure and level of granularity of LO catalogues is a well-known challenge in curriculum mapping and development, also encountered in other projects, e.g., the Erasmus+ Building Curriculum Infrastructure in Medical Education (BCIME) project [16,17]. Towards mitigation of such discrepancies, we advocate that, among other actions, implementation of bilingual LO catalogues may be useful. Albeit implementation of bilingual catalogue versions with English as a common language across the catalogues would not be a pragmatic goal for the near future, we foresee that the increasing reliability and availability of translation applications, such as DeepL, will support such undertakings over a longer term. Among various potential advantages of bilingual LO catalogues, we foresee that increased communication facilitated by a common language across LO catalogues will contribute to gradually lower heterogeneity regarding amount, structure, and level of granularity of LOs related to clinical reasoning.

We acknowledge that the use of a convenience sample of catalogues relating to involved partners and the fact that we did not apply a systematic procedure such as the Delphi technique for the derivation of the recommendations constitute some of the limitations of this work. Nevertheless, a major strength was that experts in pedagogy from several European and US universities and multiple professions within the frame of the ERASMUS+ DID-ACT consortium were involved in a joint effort for the derivation of the recommendations, in contrast with other recent nation-specific consensus statements, e.g., a US-centered by Olson et al. [23] and a UK-centered by Cooper et al. [24].

## 5. Conclusions

Based on our analysis, clinical reasoning is rarely explicitly addressed in HPE curricular policy documents. Upon systematic survey and expert consensus within the DID-ACT consortium, we herein recommend that clinical reasoning should be made explicit in national LO catalogues, including aspects of its teaching and assessment, and that collaboration aspects within and across professions, as well as with the patients, should be emphasized. We also provide examples of LOs for clinical reasoning curricula for health professions students and educators. We believe that an in-depth knowledge of the LO catalogues would support the development and enhancement of learning activities related to clinical reasoning. Finally, we anticipate that the results from the analysis presented in the current work could motivate and guide initiatives towards the implementation of LOs related to clinical reasoning in future LO catalogues.

## Figures and Tables

**Table 1:** Overview of LO catalogues in different health professions and countries.

Country	Catalogue Name	Profession	Language	Description
Europe	TUNING project	Medicine	English	Learning outcomes and competencies for undergraduate medical education in Europe
Germany	NKLM	Medicine	German	National competency-based LO catalogue for Germany
Germany	Framework for theoretical and practical teaching in nursing	Nursing	German	National framework with learning objectives and outcomes for the nursing education in Germany
Poland	Polish Ministry of Science and Higher Education	Medicine	Polish	Educational outcomes for health professions
Sweden	The Higher Education Ordinance (1993:100) Annex 2, Professional qualifications	Health professions	Swedish, English	Description of required competencies for health professions
Sweden	Entrustable professional activities (EPA)	Medicine	Swedish	Description of professional activities (EPA)
Switzerland	PROFILES	Medicine	German	A set of competency- and outcome-based LOs for medical students and faculties in Switzerland
USA	USMLE Step 2	Medicine	English	Requirements for passing the USMLE examination
USA	Adult-Gerontology Clinical Nurse Specialist Competencies	Nursing	English	Competencies for clinical nurse specialists in the US

**Table 2:** Overview of faculty development programs and frameworks.

Country	Program	Profession	Description and Structure
Germany	Requirements for a teaching certificate [Kompetenznetz Medizinlehre, Görlitz 2015]	Medicine	This framework does not refer to any specific teaching methods or contents but is divided into three levels. Basic and intermediate levels require attending a certain number of courses about teaching and learning concepts; presentation and communication techniques; assessment, reflection, and evaluation; and coaching and mentoring. The advanced level includes certain activities in the areas teaching portfolio, teaching project, teaching consultation and internship.
Germany (Heidelberg)	Master of medical education	Medicine, nursing, health sciences, therapy sciences	Postgraduate Master’s program consisting of nine modules covering topics such as curriculum development, communication in a team, teaching and assessment, educational research.
The Netherlands	Master of health professions education	Health professions	Curriculum covering eight competencies, e.g., analyze, design, communicate, and collaborate, and three roles, i.e., designer, researcher, and leader.
Sweden	Master of medical education	All healthcare professions	Master’s program preparing postgraduate students for academic leadership and educational careers.
Switzerland (Berne)	Master of medical education	All healthcare professions	Postgraduate Master’s program, advanced studies in medical education based on 12 modules covering areas such as communication, curriculum development, learning environment, and assessment.

**Table 3:** Mapping of learning objectives (LOs) related to clinical reasoning into DID-ACT categories.

Facets of Clinical Reasoning	Number of LO Catalogues	Number of LOs
Gathering, interpreting, and synthesising patient information	8	25
Developing a treatment/management plan	8	22
Ethical aspects	6	10
Decision making	6	10
Collaborative aspects of clinical reasoning	3	9
Generating differential diagnoses including defining and discriminating features	7	9
Errors in the clinical reasoning process and strategies to avoid them	5	7
Aspects of patient participation in clinical reasoning	4	6
Interprofessional aspects of clinical reasoning	3	5
Self-reflection on clinical reasoning performance and strategies for future improvement	3	4
Theories of clinical reasoning	1	1
Attitudes towards clinical reasoning teaching	0	0
Teaching, assessing, and evaluating clinical reasoning	0	0

## Data Availability

Data analyzed and generated during the present work are available in the manuscript and Supplementary Materials.

## Supplementary Materials

The following is available online at https://www.mdpi.com/article/10.3390/ijerph182111202/s1, Table S1: Chapter structure in the analyzed learning objective (LO) catalogues, and representation of clinical reasoning within chapters.
