# Supplementary material for: Clinical Reasoning Needs to Be Explicitly Addressed in Health Professions Curricula: Recommendations from a European Consortium
Source: Int J Environ Res Public Health. 2021 Oct 25;18(21):11202. doi: 10.3390/ijerph182111202 (PMC8583438; doi:10.3390/ijerph182111202)
Supplement: Supplementary file 1 [file ijerph-18-11202-s001.zip › Supplementary material.pdf]

Clinical reasoning needs to be explicitly addressed in health professions curricula: Recommendations from a European consortium

Ioannis Parodis, Lina Andersson, Steven J. Durning, Inga Hege, Jure Knez, Andrzej A. Kononowicz, Marie Lidskog, Tadej Petreski, Magdalena Szopa and Samuel Edelbring

Supplementary material

Below, we present the chapter structure in the analysed learning objective (LO) catalogues, and the representation of clinical reasoning within chapters.

Principal Relevant Objectives and Framework for Integrative Learning and Education in Switzerland (PROFILES)
Chapter structure: LOs are structured into the following three chapters: (i) CanMEDS roles, (ii) EPA, (iii) Situations as Starting Points (SPS).
Representation of LOs related to clinical reasoning: LOs related to clinical reasoning are formulated as EPAs under the clinical reasoning-related sub-chapter "Prioritise a differential diagnosis following a clinical encounter".

Polish ministry of science and higher education; educational outcomes for health professions catalogue
Chapter structure: Main LOs (n=25) are divided into three categories (knowledge, skills, social competencies). These are followed by a list of 315 specific LOs, which are grouped in the following chapters corresponding to medical domains and assigned to either knowledge or skills: (i) morphological sciences, (ii) scientific basis of medicine, (iii) preclinical sciences, (iv) social and behavioural sciences with elements of professionalism, (v) clinical non-surgical sciences, (vi) clinical surgical sciences, (vii) legal and formal aspects of medicine.
Representation of LOs related to clinical reasoning: LOs related to clinical reasoning are covered in four chapters, with a focus in "Social and behavioural sciences with elements of professionalism", and are mostly categorised as skills.

National competency-based catalogue in medicine (NKLM)
Chapter structure: Structure based on the CanMEDS roles and the following additional chapters: (i) normal structures and functions, (ii) pathogenesis and pathomechanisms, (iii) medical scientific skills, (iv) communication skills, (v) clinical skills, (vi) diagnostics, (vii) therapeutic procedures, (viii) emergency measures.
Representation of LOs related to clinical reasoning: LOs related to clinical reasoning are included in the chapters of the CanMEDS roles.

Framework for theoretical and practical teaching in nursing
Chapter structure: Framework of 11 chapters based on different nursing areas and levels. Examples are (i) promoting health and prevention, (ii) supporting people in curative processes and strengthening patient safety, (iii) acting safely in emergency situations.
Representation of LOs related to clinical reasoning: LOs related to clinical reasoning are distributed over 8 of the 11 chapters with a focus on the chapter "Supporting people in movement and self-care".

The Higher Education Ordinance
Chapter structure: The main chapters correspond to the different Bachelor and Master's programmes of healthcare and other professions.

Representation of LOs related to clinical reasoning: LOs related to clinical reasoning are included directly under the different healthcare professions.

Entrustable professional activities (EPA)
Chapter structure: Ten EPA formulated for undergraduate medical education. Each EPA is divided into sub-aspects.
Representation of LOs related to clinical reasoning: LOs related to clinical reasoning are included in three EPA.

TUNING Educational Structures in Europe
Chapter structure: Specific LOs (level 2) are assigned to more general LO chapters (level 1). The chapters are based on 12 clinical core activities, such as (i) graduates in medicine will have the ability to carry out a consultation with a patient, or (ii) graduates in medicine will have the ability to apply ethical and legal principles in medical practice.
Representation of LOs related to clinical reasoning: LOs related to clinical reasoning are distributed over five chapters, with a focus on the chapter "Graduates in medicine will have the ability to assess clinical presentations, order investigations, make differential diagnoses, and negotiate a management plan".

United States Medical Licensing Examination (USMLE) Step 2
Chapter structure: Seven chapters based on physician tasks, competencies or activities, including patient care, diagnosis, communication, professionalism, legal and ethical issues.
Representation of LOs related to clinical reasoning: LOs related to clinical reasoning are mainly included in the tasks related to "Diagnosis" and "Management", but are also included in other chapters.

Adult-Gerontology Clinical Nurse Specialist Competencies
Chapter structure: Seven chapters based on general competencies, such as (i) direct care competency, (ii) consultation competency, (iii) system leadership competency.
Representation of LOs related to clinical reasoning: LOs related to clinical reasoning are distributed over four different competencies, with a focus on "Direct care competency".
